# Ultrasound-stimulated microbubbles enhancement of fractionated radiation for tumor treatment

**DOI:** 10.1186/s12885-023-10981-5

**Published:** 2023-07-24

**Authors:** Deepa Sharma, Evan McNabb, Niki Law, Aaron Cumal, Gregory J Czarnota

**Affiliations:** 1 Physical Sciences, Sunnybrook Health Sciences Centre, Toronto, ON Canada; 2 Department of Radiation Oncology, Sunnybrook Health Sciences Centre, Toronto, ON Canada; 3 Departments of Medical Biophysics, and Radiation Oncology, Faculty of Medicine, University of Toronto, Toronto, ON Canada

**Keywords:** Ultrasound-stimulated microbubbles therapy, PC3 xenograft, Vascular disruption, Fractionated radiation therapy

## Abstract

**Background:**

Radiation therapy (XRT) causes numerous biological changes in tumor microenvironment. Radiation vascular response, due to endothelial disruption, can influence treatment outcomes in a dose-dependent manner. Ultrasound-stimulated microbubbles (USMB) have also been demonstrated to create a vascular response in the tumor microenvironment and enhance tumor response when used in combination with XRT. Single doses of 8–10 Gy are known to induce activation of acid sphingomyelinase (ASMase)-induced ceramide production, causing vascular damage. Destruction of vasculature results in endothelial apoptosis followed by tumor cell death. The effect of tumor response is known to be synergistic by 10-fold higher cell kill observed when USMB is combined with radiation.

**Methods:**

In this study, we used an USMB approach in combination with conventional low dose fractionated radiation to enhance endothelial cell responses to XRT in human PC3 prostate cancer xenograft model. Mice were divided into untreated, USMB therapy, fractionated XRT, and combined USMB therapy followed by XRT (USMB + XRT) groups. USMB therapy was delivered twice per week in the USMB-alone and combined USMB + XRT treatment groups over four weeks. Radiation treatments were delivered in fractions of 2 Gy/day (total 40 Gy in 20 fractions, BED_10_ = 48 Gy) in the XRT-alone and combined USMB + XRT groups. The treatment outcome was evaluated using histopathology, power Doppler, and immunohistochemistry assays.

**Results:**

Tumor growth assessment showed that sizes of tumors increased in the control and the single treatment groups over a treatment period of four weeks, but significantly decreased with the combined treatments of USMB + XRT. Immunohistochemical analysis indicated a statistically significant vascular disruption in mice that received treatment involving a full 4-week schedule of combined (USMB + XRT) treatments. A statistically significant increase in vascular disruption was demonstrated through CD68 and trichrome fibrosis staining. Changes in local perfusion assessed using high-frequency power Doppler imaging demonstrated attenuated blood flow in the combined group.

**Discussion and conclusions:**

This work demonstrates the efficacy of using USMB as a radiation sensitizer in a mouse model of human PC3 tumor xenograft. This radiation treatment enhancement modality has the advantage of targeting tumor vasculature with ultrasound stimulation that can be implemented prior to radiation treatment.

**Supplementary Information:**

The online version contains supplementary material available at 10.1186/s12885-023-10981-5.

## Introduction

The vascular response to ionizing radiation, specifically due to endothelial disruption has been demonstrated to be dose-dependent [1–3]. Beyond DNA damage directly to tumor cells, vascular changes are predominant at larger doses (8–16 Gy) through the activation of acid sphingomyelinase (ASMase) and ceramide production causing endothelial cell apoptosis [4]. Certain cancers in the lung, liver, and pancreas benefit from hypofractionated doses (> 2 Gy per fraction) due to dose-response relationships in order to improve local control and overall survival [5–8]. However, the use of high radiation doses per fraction is typically limited to stereotactic body radiation therapy, while standard fractionated dose regimes (1.8–2.0 Gy) are still commonly used to treat a wide range of disease sites including prostate cancers [9].

Hypofractionation differentially affects the tumor microenvironment and its vascular and oxygenation states compared to standard fractionation. Despite the potential benefits of increased ceramide production and endothelial cell disruption with increased radiation dose per fraction, hypofractionation can induce hypoxia which can lead to reduced cell kill and a reduced capacity to spare surrounding healthy tissue [2, 10]. Conversely, conventional fractionated radiation doses have been associated with reduced hypoxia [11, 12]. This type of conventional-dose regimen has been shown to preferentially kill oxygenated tumor cells, which subsequently increases tissue perfusion and improves micro-vessel stability [13]. Concomitant benefits from improved micro-vessel stability and permeability can aid in any immune response elicited by radiation. Specifically, vessel normalization can enhance the immune response as otherwise abnormal vasculature can encourage intratumoral immunosuppressive infiltrates, such as tumor-associated macrophages that promote cytokines and tumor growth [14].

In order to address the need to modify the vascular compartments within the tumor microenvironment, several combined therapies such as immunotherapy and radiation therapy (XRT) [15–17], and XRT with chemotherapy have been assessed [18]. However, optimal dose, toxicity and efficacy remain under scrutiny. Furthermore, larger pharmacological agents can have difficulty penetrating deep tumor areas that contain higher interstitial fluid pressure [19, 20]. A combined modality of radiation and ultrasound-stimulated microbubbles (USMB) has been investigated in several pre-clinical studies including in vivo tumor models from breast, prostate, and bladder cancers [21–25]. These studies investigated single-dose treatments that combined USMB with radiation. The mechanism for the radiation enhancing effect is initiated through a local mechanical disruption of the endothelial cells lining in the tumor blood vessels. This effect is then propagated through ASMase signaling pathways and ceramide production causing increased endothelial cell apoptosis leading to enhanced ischemic tumor cell death [26–30].

In the present study, we aim to build on our previous study that assessed the effects of combined radiation and USMB treatment on a prostate cancer model. A synergistic anti-tumor effect was observed by combining a single treatment of USMB and radiation [21]. In this study here, we used USMB therapy with conventional low-dose fractionated radiation to investigate the effect of treatments on a solid tumor model. The goal here was to observe vascular disruption and an immune-mediated response longitudinally that is consistent with the anti-tumor effects from USMB and larger (> 6 Gy) doses of radiation. It was hypothesized that changes to tumor growth and vascularity resulting from multi-fraction treatments will correlate with endothelial cell apoptosis. Furthermore, experimentation was carried out to explore changes in the inflammatory state and immune response in relation to the potential hypoxic or anoxic conditions created from anticipated predicted vascular destruction. The aim of combining USMB with fractionated low-dose XRT is to achieve several principles that include: targeting the radiation-sensitive cell cycle phase, reducing toxicity by allowing repair in normal cells, and allowing for the development of tumor rejection through an activated immune-response. Information acquired in this study will be the foundation for establishing and designing a future protocol for clinical trials.

## Materials and methods

### Animal handling and cell culture

All animal experiments were approved by the Sunnybrook Research Institute Animal Care Committee and compliant with national guidelines (Canadian Council on Animal Care). The prostate cancer cell line (PC3) was obtained directly from a manufacturer (ATCC, Manassas, VA, USA) and were injected into the hind leg of male Fox Chase SCID-CB-17 mice (strain code: 236, Charles River, Senneville, Canada). A total cell volume of 10^6^ in a 50 µL medium was injected subcutaneously into the hind leg of mice. Tumors were allowed to develop over four to five weeks to reach ~ 7 mm in diameter for experiments. Animals were observed in-house daily by trained veterinary staff and supportive care (analgesia and polytopic antibiotics) was given when necessary. The study here utilized any of the following humane endpoints: weight loss of more than 20% or lack of feeding, dragging tumor-bearing legs, lack of ambulation, tumors exceeding 1 cm in diameter, self-mutilation, or ulcerations exceeding 20% of the tumor area. Tumor volume was set to a threshold of 500 mm^3^ and was measured weekly along each axis by Vernier calipers. Euthanasia was performed with anesthesia and intravenously injected sodium pentobarbital (Euthanyl) immediately after reaching endpoints.

### Experimental setup

Sixty mice were divided into the following four cohort groups: untreated controls (n = 8), USMB (n = 17), fractionated XRT (n = 16), and combined treatment of USMB therapy followed by XRT (USMB + XRT) (n = 19). Humane endpoints were in the following groups: control (n = 1), USMB-alone (n = 3), XRT-alone (n = 0), combined USMB + XRT (n = 5). Six animals were found dead in cages during the tumor growth period. The total number of animals used in this study was 75.

USMB therapy was administrated twice weekly in the USMB-alone and the combined USMB + XRT treatment groups for up to four weeks. Radiation treatments were delivered in daily 2 Gy fractions five days per week, for a dose of 40 Gy in 20 fractions (BED_10_ = 48 Gy) in the XRT-alone and combined USMB + XRT groups. In the combined treatment group, radiation was delivered immediately after the USMB administration. The minimum group size to achieve a power of greater than 0.8 was determined to be n = 14, based on the minimum difference between single-dose 3% USMB and 2 Gy exposures [24].

Treatments required sedation of the animals with ketamine and xylazine as described previously [32]. A 3% (v/v) dose of microbubbles (Definity, Lantheus Medical Imaging, Billerica, MA, USA) was used. Groups receiving USMB treatment were placed in a 37 °C water bath and exposed to 500 kHz acoustic bursts (10% duty cycle wave, 3 kHz burst frequency, 570 kPa) for 50 ms. The bursts was then repeated every 2 s for a total duration of 5 min. Microbubbles were stimulated with an ultrasound beam’s peak negative acoustic pressure of 570 kPa (mechanical index of 0.8). Detailed of all equipment used have been described previously [25].

### Histopathology, CD31 and CD68 labeling

At the end of each week for four weeks, animals were sacrificed for histopathology, and tumors were excised and fixed in formalin. Several approaches were used to evaluate cellular change in response to treatments that included hematoxylin and eosin (H&E) staining for general cell morphology and Masson trichrome staining for fibrosis. Histology analysis was performed on a tumor slice section from per animal tumor. Staining for fibrosis was performed using a Richard-Allan Scientific Chroma view kit (Thermo Fisher Scientific). Cytoplasm, keratin, and muscle fibers stain red whereas mucin and collagen display blue and, black for nuclei. Quantification of trichrome staining was conducted using ImageJ (National Institutes of Health, Bethesda, MD, USA). For trichrome analysis, image masks were created from whole-slide analysis (1X or 0.8X) from the confirmed tumor areas on the H&E-stained sections, excluding skin, muscle, and necrosis. The area of positive staining was estimated relative to the total surface area examined. Vascular staining was carried out using a cluster of differentiation-31 (CD31) immunolabeling for vascular network assessment [32]. Microscopy of specimens on slides was conducted using a Leica DC100 microscope (Leica GmbH, Germany). For each stained section, five random regions of interest (ROIs) were selected (approximately 0.1 mm^2^) at 10X magnification and digitized. Using the digitized images, the number of stained blood vessels was counted within a region of interest and averaged to determine the CD31 labeling index. CD68 immunolabeling was used to partially investigate immune response. CD68 is widely used as a marker for macrophages and monocytes. High-magnification images of the stained CD68 histologic slides were subsequently obtained with a Leica DC100 microscope at 10X and the positively stained CD68 cells were counted within four-five frames per tumor section. The total area with positive CD68 staining was measured relative to the total surface area examined.

### Treatment monitoring using power doppler imaging

All tumors were imaged at the beginning of week one using a Vevo 2100 system (Fujifilm Visual Sonics, Toronto, Canada), which was considered as the baseline imaging, and at the end of each week for four weeks, to monitor change over the experimentation period. An LZ-250 transducer with a center frequency of 21 MHz was used to acquire power Doppler images at a doppler gain of 30 dB, at a pulse repetition frequency (PRF) of 2 kHz, with high persistence and a wall filter set at minimum. ROIs were selected from about 20 consecutive frames, and vascular index (VI) was calculated using MATLAB release 2015a (MATLAB, MathWorks, Natick, Massachusetts, USA) and in-house developed software (Vevolyze) [24]. The VI was calculated by dividing the number of color voxels within the ROI (representing the power Doppler signal from red blood cell backscatters) by the noncolor voxels (representing nonvascular regions).VI were averaged per image acquisition and data from images collected on week one before the start of treatments were used as the baseline, and the subsequently collected data at the end of each week after treatments were compared to the baseline. The relative change in VI was calculated using the formula VI= (VIpost—VIpre)/ VIpre. The relative change in VI for each mouse was used to assess weekly changes in tumor perfusion.

### Statistical analysis

Statistical test was performed using the one-way analysis of variance (ANOVA) followed by Tukey’s multiple comparison test using GraphPad Prism (GraphPad Software Inc, La Jolla, USA). All p-values (*p* < 0.05) were considered statistical significance and are denoted as * *p* ≤ 0.05, ** *p* ≤ 0.01, *** *p* ≤ 0.001, and **** *p* ≤ 0.0001.

## Results

### Histopathology and tumor size over the course of treatment

**Fig. 1:**
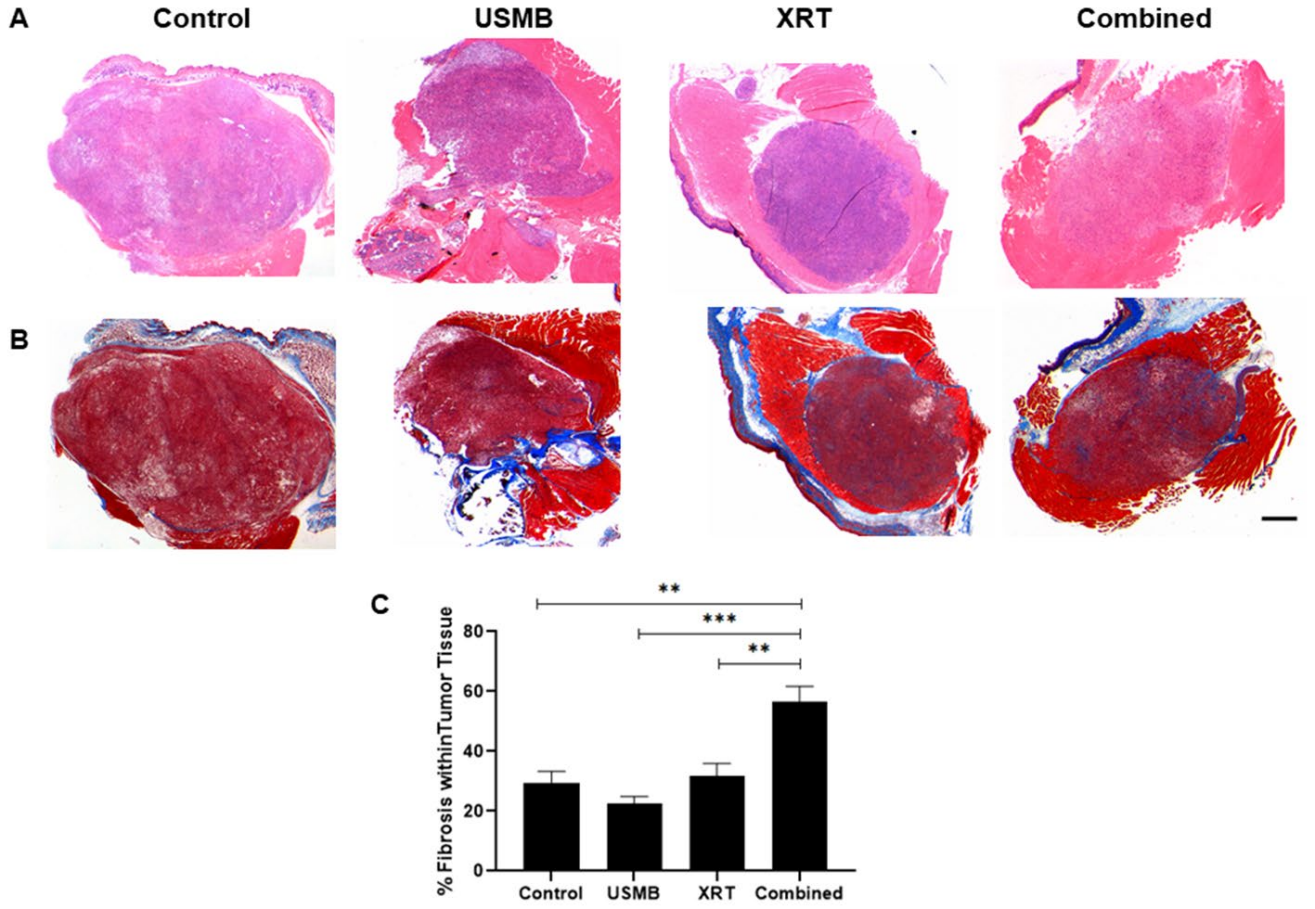
Trichrome-stained tumors show increased fibrosis after treatment. Representative (**A**) H&E and (**B**) Masson trichrome-stained slides of PC3 prostate tumors treated with USMB and/or multi-fraction XRT. Columns represent untreated tumors and tumors treated with USMB alone, XRT alone, or combined (USMB + XRT). Hematoxylin is stained in deep blue-purple color and stains nucleic acids and eosin has pink color and stains proteins nonspecifically. Blue-labeled regions (fibrosis) increased in tumors that received the combined treatment. Magnification bar equals 1 mm. (**C**) Graph quantifying the percent of fibrotic staining. Error bars represent SEM. The results showed an increment in trichrome-stained regions when USMB was combined with radiation.

Representative whole mount tumor sections of each treatment group are presented (Fig. 1 A & B). H&E-stained sections of tumors after treatment (Fig. 1 A). Tumors that remained untreated or treated with USMB only displayed minimal presence of collagen fibers with most tumor cells appearing viable. Fractionated XRT and combined USMB + XRT treated group showed increased collagen fibers (blue-colored sections) (Fig. 1B). The fibrotic staining measurements were (mean ± SEM) 29 ± 4%, 23 ± 2%, 32 ± 4%, and 56 ± 5% for the control, USMB, XRT, and combined (USMB + XRT) treatments, respectively. Tumors treated with a combination of USMB + XRT demonstrated significant increases in fibrotic staining compared to the control, USMB alone, and XRT alone groups indicating (*p* = 0.00130), (*p* = 0.0005), and (*p* = 0.0029), respectively. No significant differences were observed between USMB and XRT alone treatments, or in comparison to untreated control tumors.

**Fig. 2:**
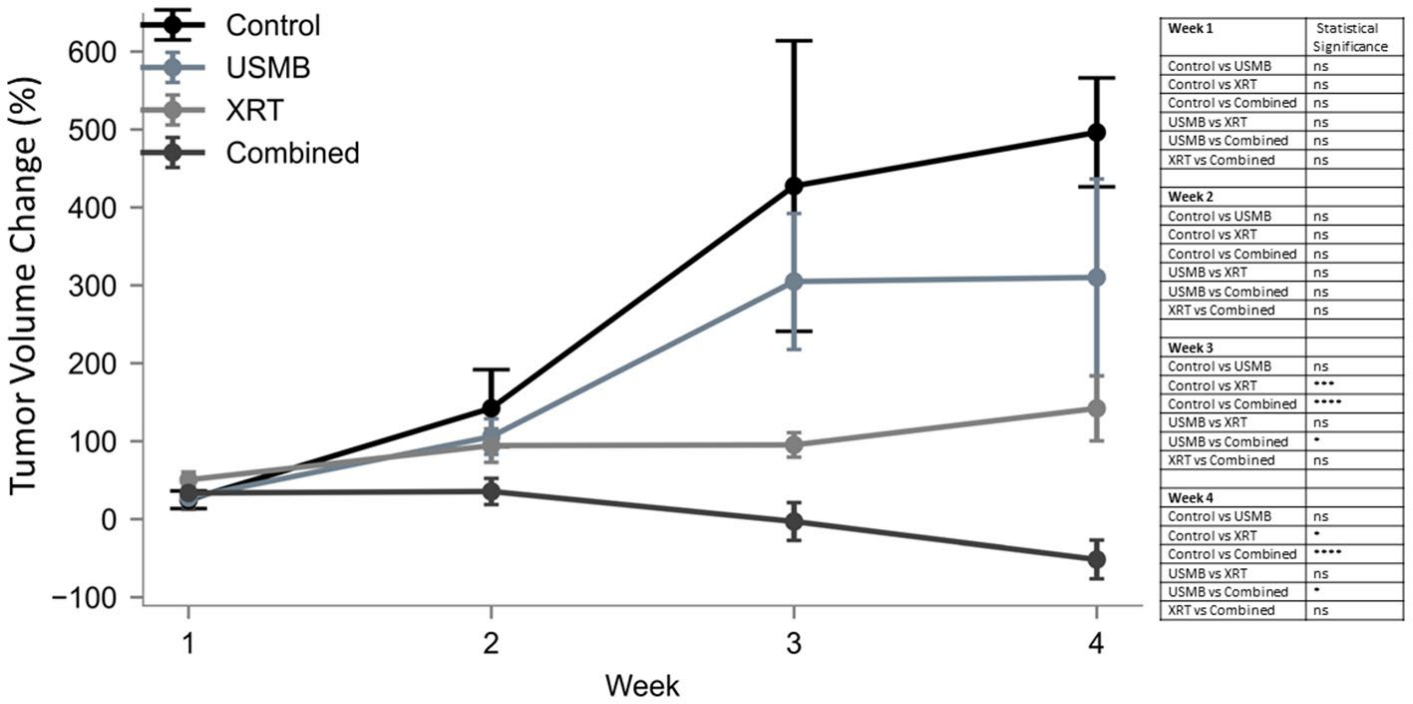
Reduction of tumor volume in mice treated with combined USMB and radiation. Weekly measurements of tumor volume (%mm^3^) relative to pretreatment baseline values are expressed as percentage change. Groups represent untreated tumors, USMB alone, multi-fraction XRT alone, and combined USMB + XRT treatments. In both non-treated controls and single-treatments, tumor size continued to increase. In contrast, under the combined treatment, a decrease was observed at the end of week one, then by a continuous decrease in weeks two, three and four. Error bars represent SEM.

All tumors were imaged prior to each treatment and at the end of each week of treatment (Fig. 2).

Tumors in the control group continued to grow rapidly with a significant increase in tumor size between weeks 1 and 4 of 471% (*p* = 0.0001) relative to their baseline values. Similarly, USMB only treated tumors also continued to grow between weeks 1 and 3 of  277% (*p* = 0.0036). Tumors treated with XRT exhibited less growth over the first three weeks of 45%, and those treated with the combined USMB + XRT therapy had negative growth (tumor shrinkage) at weeks 3 and 4. Compared to control and USMB-alone group, combined therapy demonstrated significant decreases in tumor size at weeks 3 and 4, indicating (*p* < 0.05).

**Fig. 3:**
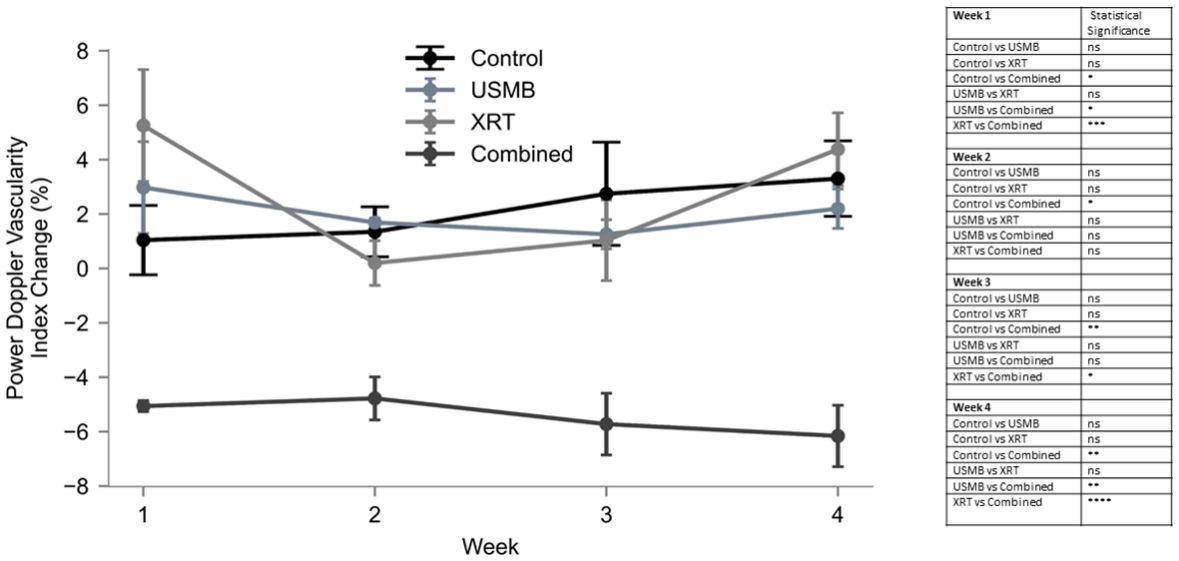
Volumetric 3D power Doppler depicting attenuated blood flow. Weekly measurements of power Doppler VI relative to pretreatment baseline values are expressed as percentage change. Groups represent untreated tumors, USMB alone, multi-fraction XRT alone, and combined USMB + XRT treatments. VI increased in non-treated control samples over the four weeks. On the contrary, a decrease in the VI was observed with the combined treatments of USMB + XRT, this decrease was significantly lower than that of the single treatments or the controls at week four. Error bars represent SEM.

VI changes were quantified and are presented (Fig. 3). The pretreatment tumor samples had VI (mean ± SEM) ranging from 1 ± 1% in week 1 to 3 ± 1% by week 4. The USMB-only group resulted in the VI changes from 3 ± 2% in week 1 to 2 ± 1% by week 4. The VI in XRT-only group changed from 5 ± 2% in week 1 to 4 ± 1% by week 4. While the VI in both group remained higher than baseline, the weekly growth changes were smaller. Finally, the combined USMB + XRT group resulted in the VI changes from -5 ± 0.2% in week 1 to -6 ± 1% by week 4. By the second week and onward, the combined treatment effect had lower VI of -5 ± 1%, -6 ± 1%, and -6 ± 1% in weeks 2, 3, and 4, respectively. There was a weekly VI attenuation in the combined group compare with control and individual treatment groups (*p* < 0.05). No weekly changes in VI were observed with individual treatment groups of USMB and XRT as compared to a control group.

### Changes in vascular density and activation of immunity

**Fig. 4:**
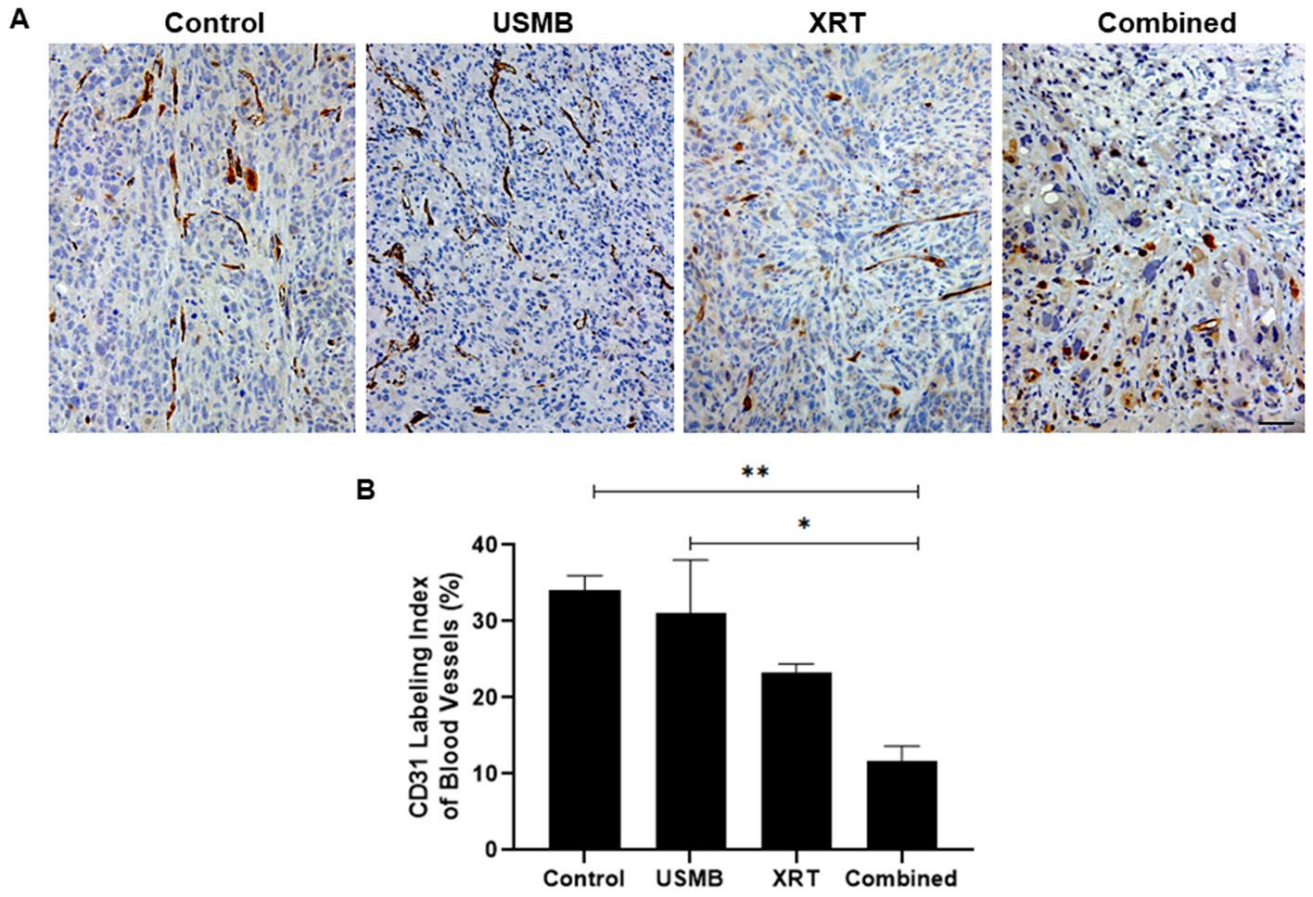
Microvascular disruption evaluated using CD31 stained tumors. (**A**) Representative high-magnification CD31 slides of PC3 prostate tumors treated with USMB alone, multi-fraction XRT alone, and combined USMB + XRT treatments. Brown color indicates CD31 staining and blue color indicates nuclear counterstaining. Scale bar equals 50 μm. (**B**) Graph quantifying the average vascular labeling of CD31 cells. Reduced vascular labeling was observed in the combined USMB + XRT treatments than in control or USMB-alone groups. Error bars represent SEM.

High magnification images of CD31 labeled stains from the subset of mice that reached four weeks of treatment are presented (Fig. 4A). The endothelial cells, visible from positive stained areas, demonstrate an increase in vascular disruption from radiation, and an approximate additive effect from the combined USMB + XRT treatment group (Fig. 4B). The CD31 staining index detected was (mean ± SEM) 34 ± 2%, 31 ± 7%, 23 ± 1%, and 12 ± 2% for control, USMB-alone, XRT-alone, and combined treatment groups, respectively. By the end of four weeks, neither the USMB-alone or XRT-alone treatment groups significantly differed from the untreated control group. The combined USMB + XRT group however, had significant differences from untreated controls and USMB-alone treatment groups with (*p* = 0.0018) and (*p* = 0.0113), respectively.

**Fig. 5:**
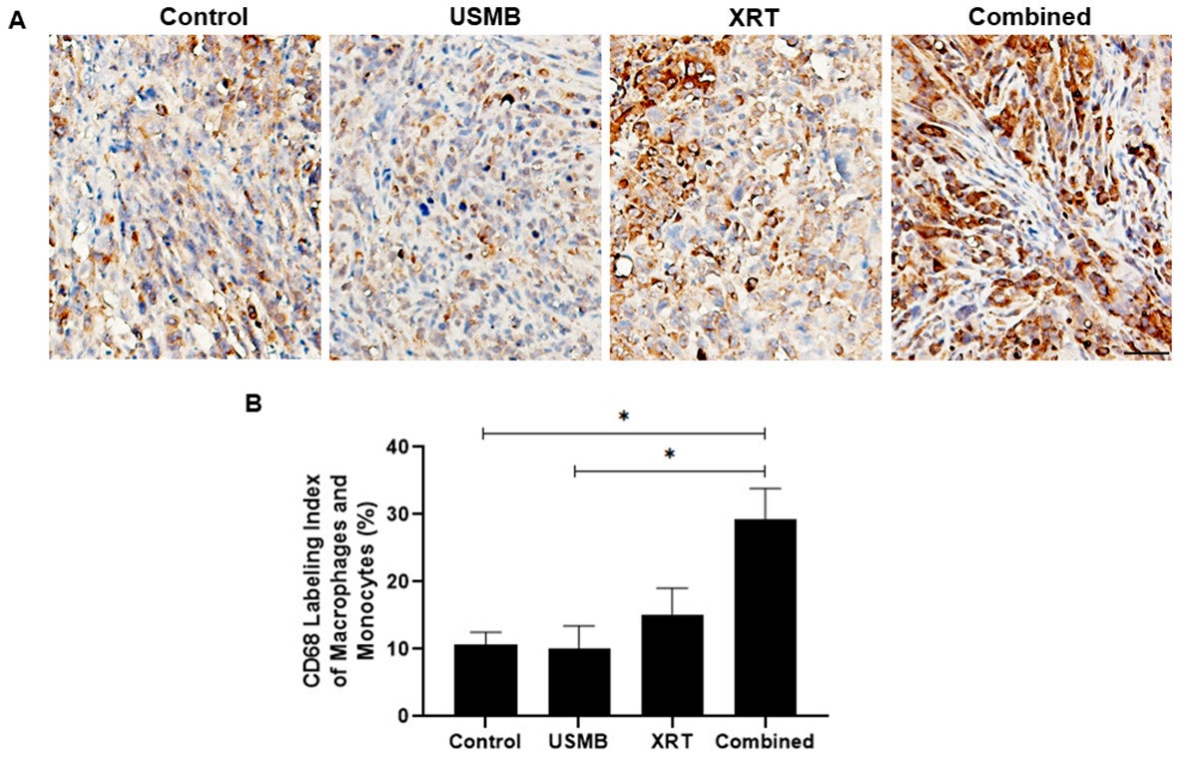
Expression of CD68 in PC3 xenograft sections. (**A**) Representative high magnification CD68 images of PC3 prostate tumors treated with USMB alone, multi-fraction XRT alone, and combined USMB + XRT treatments. High magnification views of the corresponding slides demonstrating positively stained CD68 cells were used for quantification. Brown color indicates CD68 staining and blue color indicates nuclear counterstaining. Scale bar equals 50 μm. (**B**) Graph quantifying the positive stained area of CD68 labeled cells. Combined treatment of USMB and XRT caused an increase in CD68-positive cells (brown-red labeling) compared to the control or USMB group. Labeling of macrophages and monocytes by CD68 antibody has revealed some reactivity in tumor tissues from controls, USMB and XRT treated samples. However, tumor samples treated with USMB + XRT has a significant increase in labeled macrophages and monocytes. Error bars represent SEM.

To evaluate immune response to these treatments, the same subset of mice that underwent four weeks of treatment were stained for CD68 (tumor monocytes/macrophages). Representative slides for each of the treatment conditions displays an average increase in positive staining with radiation and combined USMB + XRT (Fig. 5 A). High magnification images of CD68 stains were imaged and averaged over viable tumor. The positive staining area over all slides is quantified (Fig. 5B). The average positive stained areas were (mean ± SEM) 11 ± 2%, 10 ± 3%, 15 ± 4%, and 29 ± 5% for control, USMB-alone, XRT-alone, and combined treatment (USMB + XRT) groups, respectively. Similar to the CD31 exposures, by the end of four weeks, neither the USMB-alone or XRT-alone treatment groups significantly differed from the untreated control group. In contrast, combined USMB + XRT had significant differences compared to control and USMB-alone treatments with average increases of 19.0% (*p* = 0.0150) and 19.1% (*p* = 0.0126), respectively.

**Fig. 6:**
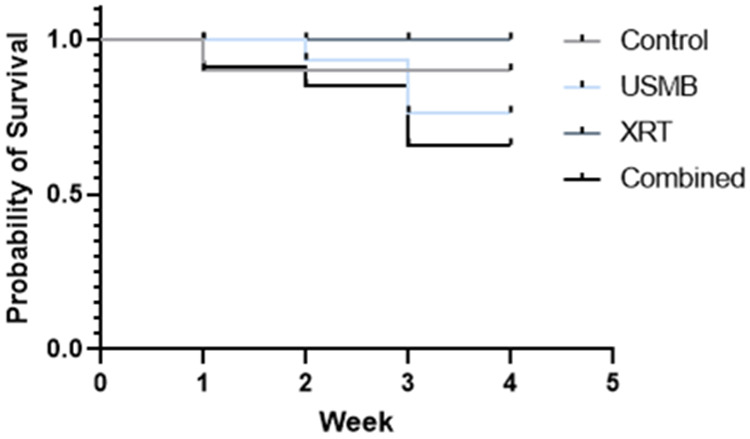
Kaplan-Meier estimates of the mice reaching humane endpoints. Groups indicate untreated tumors, USMB alone, multi-fraction XRT alone, and combined USMB + XRT treatments. Results showed the highest percent survival in combined treatments of USMB + XRT as compared to the control, USMB-only or XRT-only groups. The log-rank test did not demonstrate a significant difference by treatment group (*p* = 0.151)

Survival curves from the treatments are presented in Fig. 6. Mice survival was linked to changes in tumor size, as tumors reached end points. This approach was used to analyze survival after treatments. Under no treatment control, multiple USMB treatments, and fractionated XRT treatments, tumors continued to grow and reached end points early during treatment. However, tumors treated with multiple USMB combined with fractionated doses of XRT initially increased in size at week one, then tumor sizes started to decline after week two and continued to decline up to week four, indicating better survival. Surviving probabilities revealed no significant difference by treatment group (*p* = 0.151). Kaplan Meier estimates are presented (Fig. 6).

## Discussion

The work here demonstrates that twice weekly USMB treatment had an additive effect with fractionated XRT treatments in vivo in a mouse tumor model. For the first time, human PC3 tumor xenografts in immunocompromised SCID-CB17 mice were treated with USMB-enhanced and fractionated XRT treatment over a period of 4 weeks with an enhancement in radiation response. Histological and immunohistochemical analysis indicated a statistically significant vascular disruption in mice that received treatment over a full 4-week schedule of combined modality (USMB + XRT) treatments. The vascular disruption was associated with increased immunolabeling and fibrotic staining throughout the tumor. Furthermore, the results of this study indicate that groups receiving radiation with USMB therapy had larger decreases in perfusion, confirmed through statistical analysis of weekly changes in power Doppler imaging metrics.

The study results here agreed with previous data that suggests USMB treatment prior to XRT can cause bio-mechanical stress resulting in biochemical and morphological changes to tumor endothelial cells. Assessments using immunohistochemistry, and tumor growth arrest revealed tumor cell death indicated by increased fibrosis that was most pronounced in the mice exposed to twice weekly (3% v/v) USMB therapy combined with 40 Gy (2 Gy fractions) radiation. Previously reported tumor cell kill levels from USMB combined with single fraction 8 Gy doses of radiation on human PC3 xenografts in mice resulted in enhancements of 70% [21]. These figures were reported from in situ end-labeling (ISEL) (marker for apoptosis*)* stains, which were most apparent approximately 24 h after treatments. In the study here, mice were sacrificed at weekly intervals. The labeling of tumor cell death by ISEL is most apparent at around 24 h after the USMB + XRT treatments as was previously described [21, 22, 28], it was not detected here after several days of treatment possibly because dead cells were metabolized and replaced by fibrotic tissues. To validate this assumption, trichrome staining was carried out and it indicated the presence of more fibrotic tissues (blue color) in reduced tumors after the USMB + XRT treatments. Furthermore, USMB and XRT treatments showed a synergistic effect when combining both USMB and XRT treatments together. In order to further assess treatment efficacy and overall cell death, tumor sizes were measured at weekly intervals, where a significant reduction in tumor size was observed with specific treatments. Untreated controls and USMB-alone treatments had significant growth increases by week 3. Fractionated XRT treatments resulted in a delay of growth and combined treatments exhibited a size diminishment at four weeks of treatment.

This study differs from previous studies in the delivery of both USMB and radiation treatments. Whereas previous work focused on single-dose USMB and XRT treatments, the treatment schedule here was 40 Gy in 20 fractions (BED_10_ = 48 Gy) with the addition of twice-weekly USMB treatments. Growth analysis from previous work revealed that a 2 Gy dose (BED_10_ = 28.8 Gy) combined with USMB therapy did not significantly differ from a higher 3 Gy dose (BED_10_ = 58.5 Gy) in mice-bearing PC3 tumors [21]. It has been previously demonstrated that the enhanced sensitivity to radiation at the non-curative doses used in this study is related to ceramide production due to acoustic stress on endothelial cells. Ceramide staining after single 2 Gy + USMB treatments has been shown to be similar to single 8 Gy doses, which are expected to activate the ASMase - ceramide pathway [30]. Additionally, a study reported changes in tumor (breast MDA-MB-231) in mice over a similar observation period (28 days) after single treatments of USMB and radiation. In that work, whereas a larger radiation dose of 8 Gy in combination with USMB resulted in the greatest growth delay, single 2 Gy doses in combination with USMB also demonstrated a noticeable reduction in tumor size by four weeks [31]. Challenges facing combinational modalities include: the need to identify potential responders and to optimize efficacy, ensure reduced toxicity, which can be achieved through the optimization of timing, duration, sequence and dose. To address some of these challenges here, a low fractionated radiation dose was used with multiple USMB applications to significantly reduce toxicity. The mechanism of action is believed to be through local mechanical disruption of blood vessels, which sensitizes cells to radiation. This can possibly occur through enhanced ceramide production and signaling as was previously investigated [21, 28, 29, 32].

Power Doppler imaging assessed in this study revealed predominant changes in the VI to the periphery of the tumor in all treatments, as well as changes to the central regions in groups receiving radiation treatments. Significant decreases in the VI were observed for the combined USMB + XRT treatment group compared to USMB-alone, demonstrating there was an interaction with radiation with respect to time. A decrease in VI was observed in the combined (USMB + XRT) group starting week 1 that persisted till weeks 2, 3, and 4 as compared to control groups. The attenuation in VI in the combined group was found to be constant over the course of the treatment. This could possibly be due to the decreased size of the ROI as tumor sizes are reduced. The area of the ROI is taken into account when calculating the VI. Using a fixed smaller size ROI to analyze all the data was not an option because it would result in subjectivity when analyzing large size tumors. Work from several previous studies identified similar changes in the VI after 7 days of treatment. There, single-dose 2 Gy + USMB in mice-bearing MDA-MB-231 indicated a 43% decrease in VI 7 days after treatment, and up to 46% reductions were observed in MCA-129 fibrosarcoma tumors [24, 26, 30].

Immunolabeling with CD31 validated significant changes in the number of vascular endothelial cells. We found a significant reduction in CD31 labeling index in the combined treatment group with respect to untreated control tumors. Compared to our present study previous studies have shown a vasculature disruption of greater magnitude [24, 32]. A possible explanation of this could be the difference in observation period. Previous work observation periods ranged from 3 h to 7 days post treatment. Vascular normalization has also been shown to occur after 1–2 weeks of treatment, whereas here, endothelial destruction may be occurring in the early phases of treatment delivery. This process of predominant endothelial apoptosis has been studied in alternative cancer therapies in xenografts and human prostate cancer [33]. It is possible that vascular destruction from USMB and improved micro-vasculature stability from conventionally fractionated XRT can both occur.

Finally, this study probed the changes in immune response from each treated condition. CD68 was used to stain monocytes and macrophages. An increase in immunolabeling of macrophages in the combined USMB + XRT treatments was observed compared to untreated control samples or USMB-alone groups. In prostate cancer, increased CD68 expression has been associated with varying outcomes. There have been positive correlations to higher Gleason grades compared to benign tumors, and both negative and positive outcomes to survival [34–36]. One possibility is that the increase in macrophages here are due to a mix of both M1 and M2 subtypes of macrophages. CD68 itself is non-specific to either sub-type, though it has been shown to be from the M1 anti-tumor sub-type in higher proportions in double-stained experiments [37]. Another possibility is that the combined treatments have an increase in hypoxia-driven necrosis as has been previously demonstrated in our studies [32]. An increased expression of CD68 stained tumor-associated macrophages has demonstrated a positive correlation to angiogenesis [38], consistent with hypoxia. Overall, the increase noted was highest in necrotic areas of tumors which has been linked to areas of higher hypoxia [14, 39]. The conventionally fractionated 2 Gy radiation used here is less likely to have caused hypoxic conditions compared to hypofractionated radiation doses used elsewhere [13].

Furthermore, a survival assessment was conducted and presented in Kaplan-Meier survival curves. Combined treatments of USMB + XRT illustrated the highest percent survival when compared to control or to a treatment of USMB alone or XRT alone.

This work validated the efficacy of using fractionated schedules of acoustically-stimulated of microbubbles and whole tumor radiation to enhance human PC3 tumor to XRT in a mouse model. USMB therapy combined with XRT resulted in tumor vascular disruption and an elevated immune response. Additionally, tumor cells were replaced with fibrotic tissue. This treatment modality has the advantage of safely targeting tumor cells with ultrasound stimulation, as circulating microbubbles are also used as contrast agents in diagnostic ultrasound. Acoustic-stimulation can be chosen at a particular location and depth prior to radiation treatments.

There are a few limitations associated with this study. Even though promising results with tumor eradication were observed in this study, the application of USMB and fractionated doses in a clinical setting might be a hurdle. This treatment regimen should be optimized using larger orthotopic tumors or patient-derived xenografts. Another important point is that the use of power Doppler ultrasound imaging provides poor resolutions of the blood vessel’s structure. Techniques such as ultrasound contrast imaging or optical imaging should be incorporated to evaluate the impact of treatments on capillaries. Additionally, future work should include more specific biomarkers and molecular probes for cell death.

## Conclusion

The combinational approach of multiple USMB and low fractionated XRT doses, examined in the study here, resulted in a significant reduction in tumor sizes associated with attenuation in blood flow and tumor vascularity compared to control groups. These results support the efficacy of the new USMB approach and validate the single treatment data previously examined in both in vitro and in vivo different models. The study here is the first long-term investigation of the combinational USMB and XRT therapy that shows an effective control on solid tumor progression, and paves the way to start clinical trials.

## Electronic supplementary material

Below is the link to the electronic supplementary material.

Supplementary Material 1

## Data Availability

All the data and materials are within the manuscript.

## Declarations

### ARRIVE guidelines

The authors/manuscript follow the ARRIVE guidelines.
