# Supplementary material for: Ultrasound-stimulated microbubbles enhancement of fractionated radiation for tumor treatment
Source: BMC Cancer. 2023 Jul 24;23:693. doi: 10.1186/s12885-023-10981-5 (PMC10364378; doi:10.1186/s12885-023-10981-5)
Supplement: Supplementary file 1 — Supplementary Material 1 [file 12885_2023_10981_MOESM1_ESM.docx]

**Supplementary Information**

Table S1. Experimental design for the radiation only cohort.

| Week #1 | XRT | XRT | XRT | XRT | XRT |
| --- | --- | --- | --- | --- | --- |
| Week #2 | XRT | XRT | XRT | XRT | XRT |
| Week #3 | XRT | XRT | XRT | XRT | XRT |
| Week #4 | XRT | XRT | XRT | XRT | XRT |

Table S2. Experimental design for the USMB only cohort.

| Week #1 | USMB | ________ | ________ | ________ | USMB |
| --- | --- | --- | --- | --- | --- |
| Week #2 | USMB | ________ | ________ | ________ | USMB |
| Week #3 | USMB | ________ | ________ | ________ | USMB |
| Week #4 | USMB | ________ | ________ | ________ | USMB |

Table S3. Experimental design for the combined cohort.

| \| Week #1 \| USMB + XRT \| XRT \| XRT \| XRT \| USMB + XRT \| \| --- \| --- \| --- \| --- \| --- \| --- \| \| Week #2 \| USMB + XRT \| XRT \| XRT \| XRT \| USMB + XRT \| \| Week #3 \| USMB + XRT \| XRT \| XRT \| XRT \| USMB + XRT \| \| Week #4 \| USMB + XRT \| XRT \| XRT \| XRT \| USMB + XRT \| |
| --- | --- | --- | --- | --- | --- | --- | --- | --- | --- | --- | --- | --- | --- | --- | --- | --- | --- | --- | --- | --- | --- | --- | --- | --- |
